# SPCMLMI: A structural perturbation-based matrix completion method to predict lncRNA–miRNA interactions

**DOI:** 10.3389/fgene.2022.1032428

**Published:** 2022-11-15

**Authors:** Mei-Neng Wang, Li-Lan Lei, Wei He, De-Wu Ding

**Affiliations:** School of Mathematics and Computer Science, Yichun University, Yichun, China

**Keywords:** structural perturbation, structural consistency, matrix completion, bilayer network, lncRNA–miRNA interactions

## Abstract

Accumulating evidence indicated that the interaction between lncRNA and miRNA is crucial for gene regulation, which can regulate gene transcription, further affecting the occurrence and development of many complex diseases. Accurate identification of interactions between lncRNAs and miRNAs is helpful for the diagnosis and therapeutics of complex diseases. However, the number of known interactions of lncRNA with miRNA is still very limited, and identifying their interactions through biological experiments is time-consuming and expensive. There is an urgent need to develop more accurate and efficient computational methods to infer lncRNA–miRNA interactions. In this work, we developed a matrix completion approach based on structural perturbation to infer lncRNA–miRNA interactions (SPCMLMI). Specifically, we first calculated the similarities of lncRNA and miRNA, including the lncRNA expression profile similarity, miRNA expression profile similarity, lncRNA sequence similarity, and miRNA sequence similarity. Second, a bilayer network was constructed by integrating the known interaction network, lncRNA similarity network, and miRNA similarity network. Finally, a structural perturbation-based matrix completion method was used to predict potential interactions of lncRNA with miRNA. To evaluate the prediction performance of SPCMLMI, five-fold cross validation and a series of comparison experiments were implemented. SPCMLMI achieved AUCs of 0.8984 and 0.9891 on two different datasets, which is superior to other compared methods. Case studies for lncRNA XIST and miRNA hsa-mir-195–5-p further confirmed the effectiveness of our method in inferring lncRNA–miRNA interactions. Furthermore, we found that the structural consistency of the bilayer network was higher than that of other related networks. The results suggest that SPCMLMI can be used as a useful tool to predict interactions between lncRNAs and miRNAs.

## 1 Introduction

Non-coding RNAs (ncRNAs) are a type of RNAs that do not translate into proteins, and they were regarded transcriptional byproducts for a long time (Adelman and Egan, 2017). Along with the development of next-generation sequencing technology, researchers have found that there are only about 2% of RNA-encoding proteins in the whole human genome, while roughly up to 98% are identified as ncRNAs (Yamamura et al., 2018). However, ncRNA plays a crucial role in regulating various biological processes, such as cell cycle regulation, cell development, and tumor metastasis (Salmena et al., 2011). In human transcript expression, the length of ncRNA ranges from 22 nucleotides (nts) to hundreds of kb. Long non-coding RNAs (lncRNAs) and microRNAs (miRNAs), the two main types of ncRNAs, have attracted increasing attention for their important roles in regulating gene expression (Ambros, 2004; Bartel, 2004; Persengiev et al., 2011). miRNA is an endogenous short ncRNA molecule with a length of about 20–25 nts, which is usually involved in the gene expression regulation in post-transcription (Alvarez-Garcia and Miska, 2005; Zeng, 2006). Increasing evidence suggests that miRNAs play critical roles in many physiological and pathological processes including embryo development, tissue differentiation, cell growth, tumorigenesis, and metastasis (Liu et al., 2013; Fang et al., 2015; Sun et al., 2015). On the other side, as a kind of ncRNA with a length of more than 200 nts, lncRNAs are also widely involved in various complex biological processes such as chromatin modification, immune response and cell differentiation, growth, and apoptosis (Li et al., 2016a; Engreitz et al., 2016; Chen et al., 2018). More importantly, studies have shown that the abnormal expression of both lncRNAs and miRNAs is closely related to complex human diseases such as lung cancer, liver cancer, and gastric cancer (Huang et al., 2016; Pan et al., 2019). For example, the overexpression of lncRNA HOTAIR is related to breast cancer, colon cancer, and liver cancer; the expression of miRNA miR-145 is reduced in prostate and colon cancers (Takagi et al., 2009; Zaman et al., 2010). In recent years, with the rapid development of gene sequencing technology, more and more lncRNAs and miRNAs have been discovered, but only a small number of them have been annotated with experimental information.

A number of studies suggest that lncRNAs exert biological function roles by interacting with proteins, RNAs, and DNAs (Atianand and Fitzgerald, 2014). Such lncRNA–biomolecule interactions are very important in regulating life activities. For example, the interaction of lncRNA PVT1 with the FOXM1 protein accelerates the development of gastric cancer (Xu et al., 2017); the lncRNA loc285194 acts as a tumor suppressor by interacting with the p53 gene (Liu et al., 2013). In the past, the influence of lncRNA–miRNA interactions on the occurrence and progression of human diseases has not attracted enough attention. Recently, studies have demonstrated that lncRNA can inhibit the expression of miRNA by exerting the function of an endogenous miRNA sponge and can also act as a decoy for miRNAs to inhibit the binding of miRNA to target gene mRNA (Li et al., 2016b; Militello et al., 2017; Wang et al., 2021). Similarly, miRNAs can target a large number of protein-coding genes and lncRNAs (Paraskevopoulou and Hatzigeorgiou, 2016). For example, in glioma, knocking down the expression of lncRNA XIST can upregulate the expression of miRNA miR-152, thereby inhibiting the proliferation, invasion, and migration of cancer cells and promoting apoptosis (Yao et al., 2015). In gastric cancer, the lncRNA ANRIL regulates cell proliferation by inhibiting the expression of miRNA miR-99a and miR-499a (Zhang et al., 2014). For this reason, the lncRNA ANRIL may be used as a prognostic biomarker and new therapeutic target for gastric cancer. Although the lncRNA–miRNA regulatory network in lung cancer, colon cancer, and breast cancer has been established (You et al., 2014), there are still a large number of lncRNA–miRNA interaction regulatory networks that have not been discovered. However, identifying the interactions of lncRNAs with miRNAs through biological experiments is time-consuming, labor-compressive, and costly. In order to comprehend and deeply understand the role of lncRNA–miRNA interactions in pathophysiology and discover the potential diagnostic markers and therapeutic approaches for some specific diseases, a reasonable and effective method is urgently needed to infer the interactions of lncRNAs with miRNAs.

In recent years, many computational approaches have been introduced to identify lncRNA–biomolecule interactions, such as random forest (RF) (Wang et al., 2018), support vector machine (SVM) (Zheng et al., 2019), and non-negative matrix factorization (NMF) (Wang et al., 2022). However, methods for predicting lncRNA–miRNA interactions are still very limited. Hu et al. (2018) developed a computational method called INLMI that infers lncRNA–miRNA interactions using a matrix completion technique based on the known interaction network. Huang et al. (2018) developed a graph-based approach, named EPLMI, to predict potential interactions between lncRNAs and miRNAs. This method represents lncRNA–miRNA interaction data as a bipartite graph and uses the average of the independent prediction network based on the similarity between lncRNAs and miRNAs to calculate the final prediction network. Wong et al. (2020) constructed a lncRNA–miRNA bipartite network and used linear neighbor representation to infer the potential interactions between lncRNAs and miRNAs (LNRLMI). Xu et al. (2021) developed a structural perturbation method to predict potential lncRNA–miRNA interactions, but this method only considered the expression profile information on lncRNAs and miRNAs when constructing the lncRNA similarity network and miRNA similarity network. In addition, nonnegative matrix factorization (NMF) is an efficient method and has been successfully used for data representation (Lee and Seung, 1999). The purpose of NMF is to approximate a matrix by the product of two low-rank nonnegative matrices. Pauca et al. (2006) proposed a constrained nonnegative matrix factorization (CNMF) method for data representation, which uses regularization constraint terms in NMF to mine the intrinsic geometry of the data space. Wang et al. (2020) proposed a graph regularized nonnegative matrix factorization method for inferring interactions of lncRNAs with miRNAs (GNMFLMI). Most of the previous methods aimed to improve the accuracy of prediction but ignored the range of lncRNA–miRNA interactions that can be predicted.

In this paper, we proposed a novel computational model, called SPCMLMI, to infer potential interactions of lncRNAs with miRNAs based on matrix structural perturbation. More specifically, we constructed a duplex network and randomly selected partial observed links from a duplex network to construct the perturbation set. Then, perturbing the remaining links, a perturbed adjacency matrix can be obtained by first-order approximation. Finally, we rank the unobserved links according to the scores of the perturbed matrix. In principle, the miRNAs with higher scores in each column are more likely to interact with the corresponding lncRNA. The proposed method has the following advances: 1) we built a bilayer network by integrating the confirmed lncRNA–miRNA interaction network, the lncRNA similarity network, and the miRNA similarity network, which can fuse more effective information to improve the prediction performance. 2) Considering that there is no prior knowledge on network organization in the structural consistency index, the structural consistency index was used to evaluate the link predictability of the lncRNA–miRNA interaction network. The results suggest that the consistency of the bilayer network is superior to other related networks. Under five-fold cross validation, SPCMLMI achieved AUC values of 0.8984 and 0.9891 on two different datasets, respectively, which outperformed other comparative methods. In addition, compared to the correlation network, the bilayer network also showed the best performance. The experimental results suggest that SPCMLMI can effectively infer lncRNA–miRNA interactions and provide valuable information for biomedical research.

## 2 Materials and methods

### 2.1 Datasets

For investigating the potential interactions of lncRNAs with miRNAs, we downloaded the lncRNASNP database from http://bioinfo.life.hust.edu.cn/lncRNASNP as the baseline dataset (Gong et al., 2015). In the lncRNASNP database, there are 8,091 laboratory study-verified records of known interactions between lncRNAs and miRNAs which were collected from 108CLIP-Seq datasets. After deleting the invalid lncRNAs and miRNAs and the duplicated records, we obtained 5,118 valid lncRNA–miRNA interaction pairs used as the benchmark data in our study, including 780 lncRNAs and 275 miRNAs. In order to better describe the lncRNA–miRNA interactions, we constructed the lncRNA–miRNA adjacency matrix ${LM}^{m\times n}$ , where m and n represent the number of lncRNAs and miRNAs, respectively. The element value LM(i,j) of the adjacency matrix is assigned 1 if lncRNA $l_{i}$ is related to miRNA $m_{j}$ ; otherwise, it is 0.

### 2.2 Method overview

**FIGURE 1:**
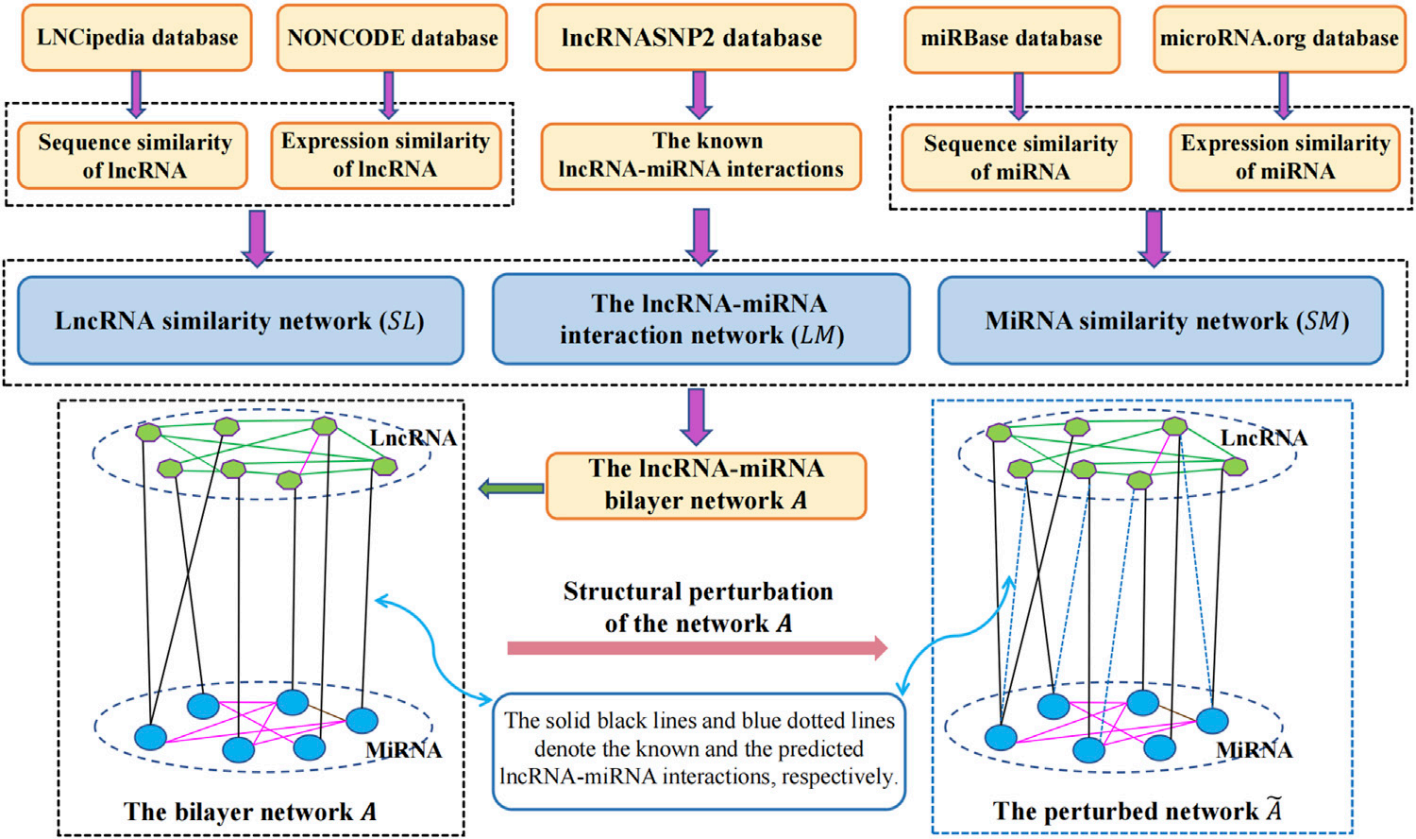
Flowchart of the prediction process of SPCMLMI.

In this study, to infer the undiscovered interactions of lncRNAs with miRNAs, we proposed a link prediction approach called SPMCLMI, which achieved matrix completion based on the structural perturbation of the bilayer network. The overall process of SPMCLMI is given in Figure 1. First, we calculated the expression similarity network using Pearson’s correlation coefficient based on the expression profile of lncRNAs and miRNAs, respectively. Considering that some RNAs have no expression similarity, we calculated the second type of similarity network for RNAs based on sequence information. According to the aforementioned two similarities, the integrated similarity network for lncRNAs and miRNAs was constructed, respectively. Second, we constructed the bilayer network A based on the lncRNA similarity network SL , miRNA similarity network SM , and lncRNA–miRNA interaction network LM . Finally, the scores of all unobserved lncRNA–miRNA links were obtained by structural perturbation.

### 2.3 Construction of the lncRNA–miRNA bilayer network

The lncRNA–miRNA bilayer network consists of three networks, namely, the known lncRNA–miRNA interaction network, lncRNA similarity network, and miRNA similarity network.

In this work, for calculating the similarities among RNAs, two different types of lncRNA/miRNA information were collected to construct lncRNA and miRNA similarity networks, including expression profiles and sequence information on nucleotides. Based on the hypothesis that functionally similar miRNAs/lncRNAs tend to interact more with a cluster of lncRNAs/miRNAs which share similar functions, Pearson’s correlation coefficient (PCC) has been widely utilized to calculate the similarity of ncRNAs (Wang et al., 2020). Here, we used PCC to calculate the first kind of similarity based on the expression profiles of lncRNAs and miRNAs. For each lncRNA, the expression profiles can be collected from NONCODE (Bu et al., 2012), while the expression profiles of each miRNA can be obtained from the microRNA.org database (Betel et al., 2008). Therefore, given two expression profiles of lncRNA $l_{i}$ and lncRNA $l_{j}$ ( $X_{l}=\left\{x_{l1},x_{l2},\cdots ,x_{lt}\right\}$ and $Z_{l}=\left\{z_{l1},z_{l2},\cdots ,z_{lt}\right\}$ ), the similarity score is defined as follows:

$$PS\_L\left(l_{i},l_{j}\right)=\frac{\left|\overset{h}{\underset{i=1}{\sum }}\left(x_{li}-{\bar{X}}_{l}\right)\left(z_{li}-{\bar{Z}}_{l}\right)\right|}{\sqrt{\overset{t}{\underset{i=1}{\sum }}{\left(x_{li}-{\bar{X}}_{l}\right)}^{2}}\sqrt{\overset{t}{\underset{i=1}{\sum }}{\left(z_{li}-{\bar{Z}}_{l}\right)}^{2}}}$$ (1)

where $\bar{X_{l}}$ and $\bar{Z_{l}}$ represent the average value of $X_{l}$ and $Z_{l}$ , respectively. h is the number of attributes of the expression profile. In general, a larger $PS\_L\left(l_{i},l_{j}\right)$ represents a more similar expression between lncRNAs $l_{i}$ and $l_{j}$ . Similarly, the expression similarity PS_M of each pair of miRNAs can be also calculated.

The second type of RNA similarity was measured based on the sequence information on nucleotides. The sequence information on lncRNAs and miRNAs was obtained from the LNCipedia database (Volders et al., 2013) and miRBase database (Kozomara and Griffiths-Jones, 2014), respectively. Given the sequence information on lncRNAs, the sequence similarity $QS\_L\left(l_{i},l_{j}\right)$ between lncRNA $l_{i}$ and lncRNA $l_{j}$ can be calculated using the Needleman–Wunsch pairwise sequence alignment (Cock et al., 2009). Considering that a few lncRNAs and miRNAs have no corresponding expression profiles, we integrated two different types of similarity networks so as to complement the missing similarity information. Specifically, the average values of the expression profile similarity and sequence similarity were used to denote the comprehensive similarity of lncRNAs and miRNAs. The final lncRNA similarity was calculated as follows:

$$SL\left(l_{i},l_{j}\right)=\frac{PS\_L\left(l_{i},l_{j}\right)+QS\_L\left(l_{i},l_{j}\right)}{2}$$ (2)

By applying the same method for miRNAs, the final similarity of miRNA $m_{i}$ and miRNA $m_{j}$ was calculated as follows:

$$SM\left(m_{i},m_{j}\right)=\frac{PS\_M\left(m_{i},m_{j}\right)+QS\_M\left(m_{i},m_{j}\right)}{2}$$ (3)

Finally, by integrating the lncRNA similarity network SL , miRNA similarity network SM , and the lncRNA–miRNA interaction network LM , we constructed a lncRNA–miRNA bilayer network and denoted it by the matrix $A\in R^{N\times N}$ as follows:

$$A=\left[\begin{array}{cc} SL & LM\\ {LM}^{T} & SM \end{array}\right]$$ (4)

The sizes of SL , SM , and LM are m×m , n×n , and m×n (m=780,n=275) , respectively. N is the total number of lncRNAs and miRNAs.

### 2.4 Structural consistency index

In 2015, Lü et al. (2015) developed a new approach named structural consistency for quantifying the link predictability of complex networks. This approach mainly considers the consistency of the structural features of existing networks before and after randomly removing a small set of associations. In this study, we used structural consistency to evaluate the lncRNA–miRNA bilayer network A . The weights of the bilayer network are LM values, SL values, and SM values, respectively. We used a graph *G (T, E,* and *W)* to represent the weighted bilayer network A , where T denotes the set of nodes consisting of lncRNA and miRNA nodes, E denotes the set of edges, and W denotes the weights of each edge. We randomly select partial edges from the bilayer network to construct a perturbation set $E^{p}$ , and the remaining edges are represented as $E^{r}$ . The perturbation set $E^{p}$ and the remaining of the edge set $E^{r}$ are represented as the matrices $A^{p}$ and $A^{r}$ , respectively. In fact, the matrices of $A=A^{p}{+A}^{r}$ , $A^{p}$ , and $A^{r}$ are real symmetric. Therefore, we can diagonalize the matrix $A^{r}$ as follows:

$$A^{r}=\overset{N}{\underset{k=1}{\sum }}\lambda_{k}x_{k}x_{k}^{T},$$ (5)

where $\lambda_{k}$ denotes the eigenvalue of $A^{r}$ and $x_{k}$ denotes the corresponding orthogonal and normalized eigenvector. Based on the first-order approximation principle that keeps the eigenvectors unchanged, $E^{p}$ is used as a perturbation of the network $A^{r}$ to obtain a perturbed matrix. The eigenvalues of the matrix may be degenerate or non-degenerate. Therefore, we analyzed the cases with and without repeated eigenvalues separately. The first case is that there are no repeated eigenvalues. After perturbation, the eigenvalue and the corresponding eigenvector change from $\lambda_{k}$ and $x_{k}$ to $\lambda_{k}+∆\lambda_{k}$ and $x_{k}+∆x_{k}$ , respectively. According to the definition of eigenfunction, we obtain the following equation:

$$\left(A^{r}+A^{p}\right)\left(\;x_{k}+∆x_{k}\right)=\left(\lambda_{k}+∆\lambda_{k}\right)\;\left(x_{k}+∆x_{k}\right).$$ (6)

Here, left-multiplying $x_{k}^{T}$ for Eqn. 6 and ignoring the second-order terms $x_{k}^{T}A^{p}∆x_{k}$ and $∆\lambda_{k}x_{k}^{T}∆x_{k}$ , the increment of the eigenvalue can be expressed as follows:

$$∆\lambda_{k}\approx \frac{x_{k}^{T}A^{p}x_{k}}{x_{k}^{T}x_{k}}.$$ (7)

The remaining eigenvectors are unchanged, the eigenvalue $\lambda_{k}$ of $A^{r}$ in Eqn. 5 is replaced by the perturbed eigenvalue $\lambda_{k}+∆\lambda_{k}$ , and we can obtain the perturbed matrix as follows:

$$\tilde{A}=\overset{N}{\underset{k=1}{\sum }}\left(\;\lambda_{k}+∆\lambda_{k}\right)x_{k}x_{k}^{T},$$ (8)

where $\tilde{A}$ can be seen as a linear approximation of the network *A*.

The second case is that the adjacency matrix has repeated eigenvalues. Here, we use $\lambda_{ki}$ to represent the eigenvalues of $A^{r}$ , the index k is the kth eigenvalue, and the index i is M -related eigenvectors corresponding to the same eigenvalue. It is worth noting that for the eigenvectors corresponding to the same eigenvalue, their linear combination is still the eigenvector of the corresponding matrix. Studies have confirmed that repeated eigenvalues are associated with the symmetric graphs and their automorphisms in networks. If perturbing the network $A^{r}$ , the nodes’ symmetry will be improved, so the degenerate eigenvalues can be converted into non-degenerate eigenvalues by perturbing the network. Therefore, we can use the non-degenerate eigenvalue case to modify this case. Given the eigenvectors ${\tilde{x}}_{ki}=\sum_{j=1}^{M}\beta_{kj}x_{kj}$ , the eigenfunction can be formularized as follows:

$$\left(A^{r}+A^{p}\right){\tilde{x}}_{ki}=\left(\lambda_{ki}+∆{\tilde{\lambda }}_{ki}\right)\;{\tilde{x}}_{ki}$$ (9)

giving us

$$∆{\tilde{\lambda }}_{ki}\overset{M}{\underset{j=1}{\sum }}\beta_{kj}x_{kj}=\overset{M}{\underset{j=1}{\sum }}\beta_{kj}{A^{p}x}_{kj}.\;$$ (10)

Thereafter, left multiplying $x_{kp}^{T}$ in Eqn. 10 ( p=1,2,⋯⋯,M ),

$$∆{\tilde{\lambda }}_{ki}\beta_{kp}=\overset{M}{\underset{j=1}{\sum }}\beta_{kj}x_{kq}^{T}{A^{p}x}_{kj}.$$ (11)

The aforementioned Eqn. 11 can be written in the matrix form as follows:

$$HB_{k}=∆{\tilde{\lambda }}_{ki}B_{k},$$ (12)

where $B_{k}$ is the column vector of $\beta_{kj}$ , H is an M×M matrix, and $H_{qj}=x_{kq}^{T}{A^{p}x}_{kj}$ . Finally, $∆{\tilde{\lambda }}_{ki}$ and $B_{k}$ can be obtained based on the eigenfunction Eq. 12, and the perturbed matrix is calculated by replacing $∆\lambda_{k}$ and $x_{k}$ with $∆{\tilde{\lambda }}_{k}$ and ${\tilde{x}}_{k}$ in Eq. 8, respectively. In other words, we transformed the case where the adjacency matrix has degenerate eigenvalues into the case with non-degenerate eigenvalues.

The eigenvectors of a matrix can be used to measure the network structure. In general, if the eigenvectors of the perturbed matrix $\tilde{A}$ and the original adjacency matrix A are almost the same, it means that the perturbation set does not sharply change the network structure of the matrix. If so, the network has high structural consistency. Therefore, given a network A , we perturbed $A^{r}$ by $E^{p}$ to calculate the perturbed matrix $\tilde{A}$ based on Eq. 8. In order to measure the structural consistency, all of the edges in ${A-E}^{r}$ and the unobserved edges were sorted in descending order based on the values of the perturbed matrix $\tilde{A}$ . $E^{l}$ denotes the top-*L* scores in $\tilde{A}$ , and l denotes the number of edges in the perturbation set $E^{p}$ . Structural consistency δ is defined as follows:

$$\delta =\frac{\left|E^{l}\cap E^{p}\right|}{l}$$ (13)

where $\left|E^{l}\cap E^{p}\right|$ denotes the number of shared edges between $E^{l}$ and $E^{p}$ . For example, we removed the edges (1,6), (2,7), (2,9), (3,8), and (4,12) to construct a perturbation set $E^{p}$ , ( i.e., l=5 ). After perturbation, the top-*L* edges in $E^{l}$ were (1,6), (2,9), (4,7), (4,8), and (4,12). Thus, the structural consistency is δ=3/5=0.6.

In this work, the structural consistency of four related networks was calculated, including the lncRNA–miRNA interaction network LM *,* LM+SL , LM+SM , and lncRNA–miRNA bilayer network A . During perturbation, we randomly selected 10% of the total edges E to construct the perturbation set. As shown in Table 1, the lncRNA–miRNA bilayer network A achieved the highest structural consistency, suggesting that the inclusion of more information in the network can improve the structural consistency. Moreover, the structural consistency δ of the LM+SL network is higher than that of the LM+SM network, which shows that there is more helpful information on LM+SL than LM+SM . Therefore, we can improve the predictability by effectively integrating information from different sources.

**TABLE 1:** Structural consistency of four related networks on the lncRNASNP dataset.

Network	*LM*	*LM + SM*	*LM + SL*	Bilayer network (A)
Structural consistency	0.2732 ± 0.0079	0.4274 ± 0.0054	0.5247 ± 0.0008	**0.5617 ± 0.0008**

## 3 Results

### 3.1 Evaluation metrics

To systematically investigate the performance of SPCMLMI, we implemented the five-fold cross validation experiments on the lncRNASNP dataset and compared it with other methods. In the framework of five-fold cross validation, the observed lncRNA–miRNA interaction pairs were randomly divided into five equally sized subsets. For these subsets, each subset was taken in turns as the test set for validating the model, while the rest of the four subsets served as the training set. More specifically, for the lncRNA–miRNA bilayer network A , we infer potential interactions between lncRNA and miRNA by using structural perturbation. The originally known lncRNA–miRNA interactions were partitioned into five groups. One of them was used as the probe sample and the remaining other groups together with SL and SM composed the training set. Then, a fraction of links was removed from the training set to be used as the perturbation set. Finally, we can obtain the perturbed matrix $\tilde{A}$ by Eqn. 8. Moreover, to reduce the bias caused by perturbation set selection, the final predicted matrix $\tilde{A}$ was calculated by averaging t independent perturbations.

The receiver operating characteristic (ROC) curve is an important metric for studying the generalization performance of a learner. We can plot the ROC curve by setting different thresholds for a false positive rate (FPR) and true positive rate (TPR). The area under the ROC curve (AUC) is widely used to estimate the performance of models, which follows the principle of the larger the better. If AUC = 0.5 represents random performance, AUC = 1 represents perfect performance. The FPR and TPR are calculated as follows:

$$FPR=\frac{FP}{TN+FP}$$ (14)

$$TPR=\frac{TP}{TP+FN}$$ (15)

Furthermore, to measure the performance of the proposed model from multiple perspectives, a range of evaluation indicators called specificity (Spe.), precision (Pre.), sensitivity (Sen.), accuracy (Acc.), and F1-score are defined as follows:

$$Specificity=\frac{TN}{TN+FP}$$ (16)

$$Precision=\frac{TP}{TP+FP}$$ (17)

$$Sensitivity=\frac{TP}{TP+FN}$$ (18)

$$Accuracy=\frac{TP+TN}{TP+TN+FP+FN}$$ (19)

$$F1-Score=\frac{2*Pre.*Sen.}{Pre.+Sen.}$$ (20)

where TP and TN are the number of true positive and true negative samples, respectively, and FP and FN are the number of false positive and false negative samples, respectively.

**FIGURE 2:**
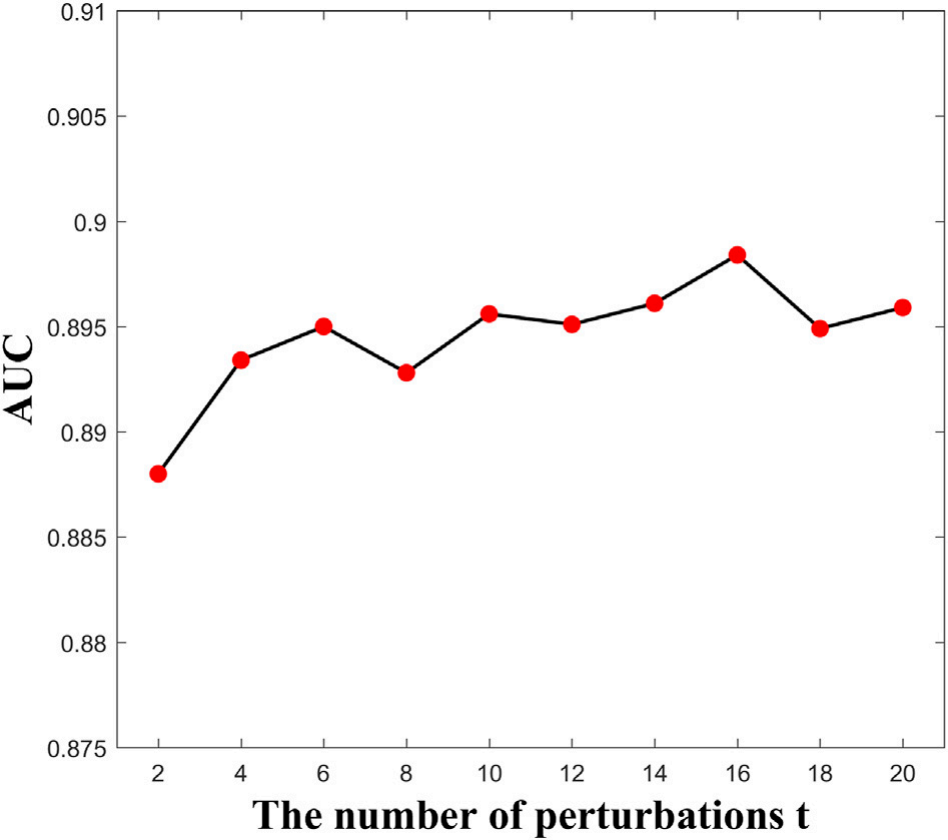
AUC values versus parameter *t*.

Here, the parameter t denotes the number of perturbations. We investigate how the parameter t∈{2,4,6,……,18,20} influenced the performance of the bilayer network constructed in Section 2.3. The effect of the number of perturbations t on the prediction performance is shown in Figure 2. Each point denotes the average of the AUC value under five-fold cross validation. The performance is optimal when t=16 . It is worth noting that all parameters in the compared methods are default.

### 3.2 Prediction performance of a structural perturbation-based matrix completion method to predict lncRNA–miRNA interactions

**FIGURE 3:**
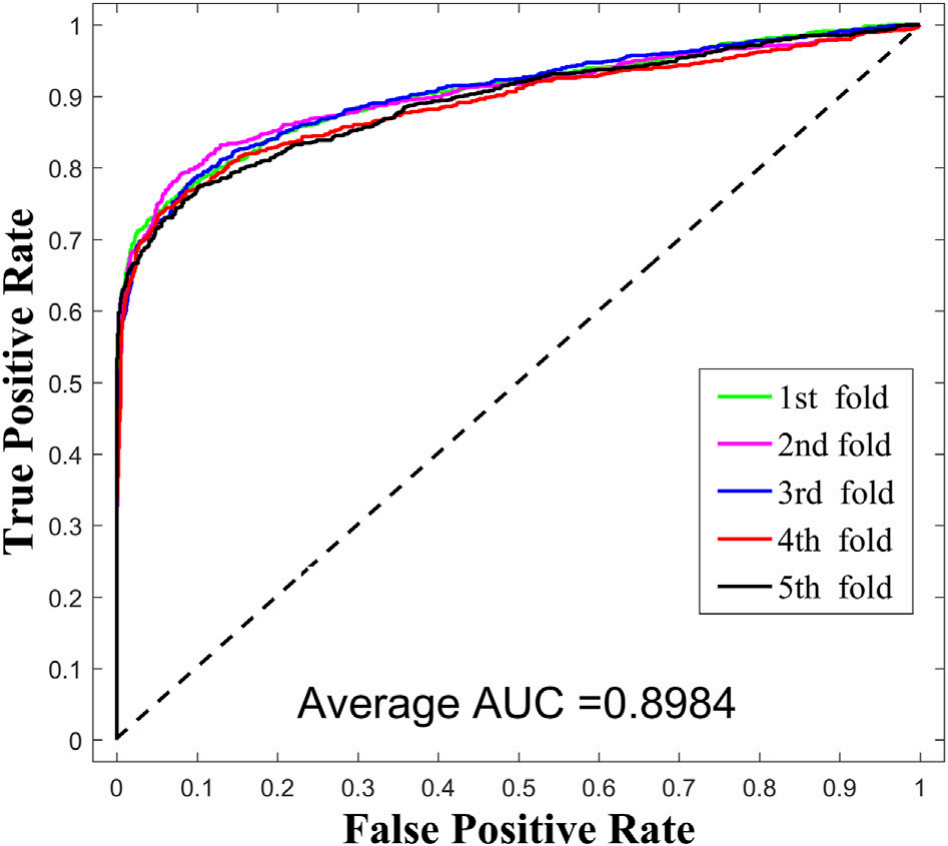
Performance results of SPCMLMI using the bilayer network.

In this work, we compared SPMCLMI with some previous studies, including INLMI (Hu et al., 2018), EPLMI (Huang et al., 2018), KATZ (Chen et al., 2017), LMF (Koren, 2008), NMF (Lee and Seung, 1999), CNMF (Pauca et al., 2006), and GNMFLMI (Wang et al., 2020). The KATZ measure, as an effective network-based link prediction algorithm, has been widely used in bioinformatics. The latent factor model (LFM) is a recommendation system algorithm, which aims to find the relationship matrix between lncRNA/miRNA and the latent factor and then takes the product of the aforementioned two matrices as the score matrix for the interaction between lncRNAs and miRNAs. As shown in Figure 3 and Table 2, we use the AUC as an evaluation indicator of model performance. The SPMCLMI model achieved the best performance among eight compared methods on the lncRNASNP dataset. Specifically, the average AUC values of SPMCLMI, INLMI, EPLMI, LMF, KATZ, NMF, CNMF, and GNMFLMI were 0.8984, 0.8517, 0.8402, 0.8257, 0.7435, 0.8316, 0.8535, and 0.8894, respectively. The AUC values of SPMCLMI were 4.67%, 5.82%, 7.27%, 15.49%, 6.68%, 4.49%, and 0.9% higher than those of the aforementioned seven computational approaches, respectively. The experimental results demonstrated that SPMCLMI is an efficient method in inferring large-scale lncRNA–miRNA interactions.

**TABLE 2:** Average AUC values achieved among different methods under five-fold cross validation on the lncRNASNP dataset.

Method	SPCMLMI	INLMI	EPLMI	KATZ	LMF	NMF	CNMF	GNMFLMI
AUC	0.8984	0.8517	0.8402	0.7435	0.8257	0.8316	0.8535	0.8894

In addition, we calculated the values of specificity, precision, sensitivity, accuracy, and F1-score under five-fold cross-validation of SPCMLMI on the lncRNASNP dataset. As shown in Table 3, the average Acc. of SPCMLMI was 84.33%, and the Acc. under the five-fold cross-validation experiment was 84.36%, 85.45%, 84.38%, 84.03%, and 83.45%, respectively, while the standard deviation is only 0.73%. In terms of indices such as Spe., Pre., Sen., and F1-score, the proposed method obtained average values of 92.34%, 90.94%, 76.33%, and 82.97%, and their standard deviation was 1.90%, 1.90%, 2.10%, and 0.91%, respectively. These results proved that the proposed method is very suitable for predicting lncRNA–miRNA interactions.

**TABLE 3:** Sep., Sen., Pre., Acc., and F1-score values achieved by SPCMLMI on the lncRNASNP dataset.

Fold	Sep. (%)	Sen. (%)	Pre. (%)	Acc. (%)	F1-score (%)	AUC (%)
1st	93.75	75.00	92.31	84.36	82.76	90.36
2nd	92.19	78.71	90.97	85.45	84.40	90.29
3rd	91.21	77.54	89.82	84.38	83.23	90.41
4th	94.63	73.44	93.18	84.03	82.14	88.90
5th	89.92	76.95	88.44	83.45	82.30	89.25
Average	92.34 ± 1.90	76.33 ± 2.10	90.94 ± 1.90	84.33 ± 0.73	82.97 ± 0.91	89.84 ± 0.71

**FIGURE 4:**
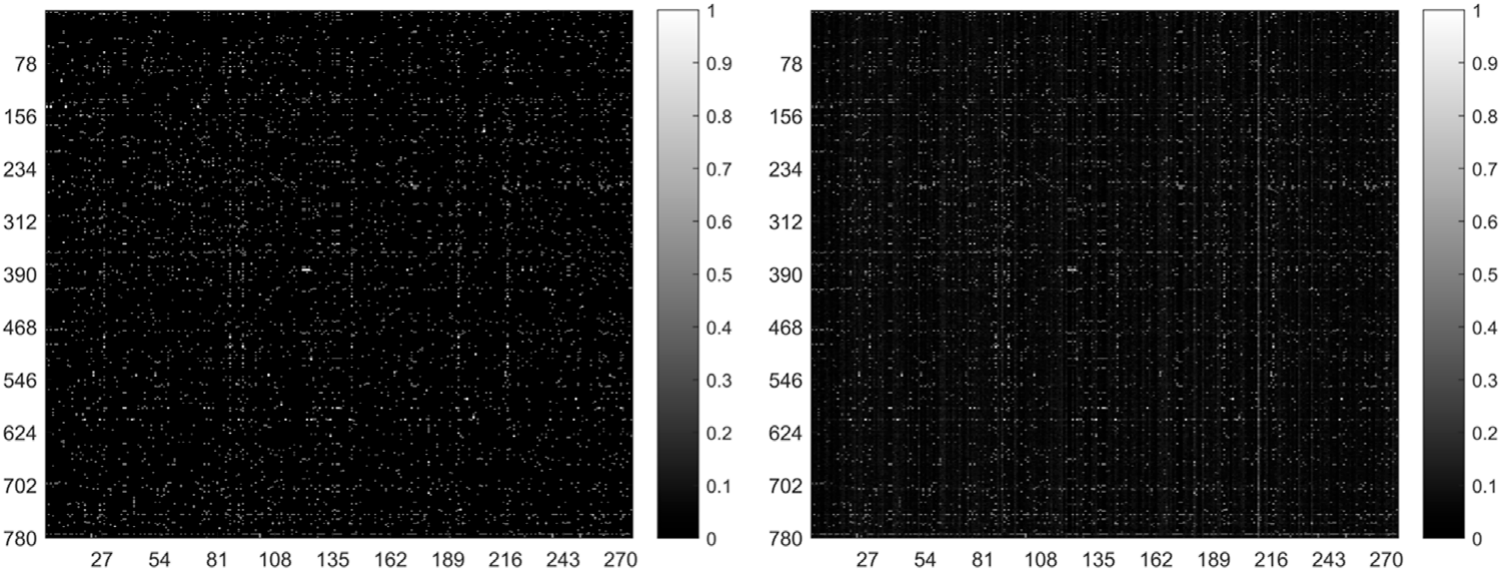
Original lncRNA–miRNA interaction adjacency matrix (left) and the result matrix (right).

In general, the predicted results obtained from the top-ranked are more convincing than others. In other words, in the predicted matrix, larger values suggest that the lncRNAs are more likely to interact with the corresponding miRNAs. Here, all verified lncRNA–miRNA interactions were used as the training sample, and the number of correctly recovered known interactions was used to judge the effectiveness of the model. Usually, the model is considered more effective if more true interactions are retrieved from the top-ranked parts. The original lncRNA–miRNA interaction adjacency matrix and the result matrix are shown in Figure 4. From Figure 4, we can visually observe that our proposed model successfully retrieved the vast majority of interactions from all the 5,118 known interactions, suggesting that SPCMLMI is an effective approach in retrieving known lncRNA–miRNA interactions with a lower false positive rate.

### 3.3 Comparison with the other three related networks

To further investigate the impact of various networks’ information on prediction performance of SPCMLMI, we compared the performance of four related networks including the bilayer network A *,* LM+SL , LM+SM , and LM in inferring interactions between lncRNAs and miRNAs. In Section 2.4, we calculated the structural consistency of the aforementioned four networks. Compared with the other three networks, the bilayer network A obtained the highest structural consistency. Table 4 shows the AUC values of four related networks under the five-fold cross-validation experiment. It is obvious from Table 4 that the bilayer network A achieved the best performance among the four cases. The AUC values of the bilayer network A *,* LM+SL network, LM+SM network, and LM network were 0.8984, 0.8468, 0.8315, and 0.8209, respectively. The experimental results show that the performance and structural consistency of these related networks tend to be consistent. In addition, the AUC values of the bilayer network *A* were 5.16%, 6.69%, and 7.75% higher than those of the other three networks, suggesting adding similarity networks *SL* and *SM* can effectively improve the prediction performance of lncRNA–miRNA interactions.

**TABLE 4:** AUC values of four related networks by using SPCMLMI on the lncRNASNP dataset.

Network	Five-fold cross validation					Average (std)
	1st	2nd	3rd	4th	5th	
LM	0.8361	0.8124	0.8201	0.8103	0.8257	0.8209 (0.0105)
LM + SM	0.8458	0.8312	0.8401	0.8228	0.8175	0.8315 (0.0117)
LM + SL	0.8433	0.8568	0.8474	0.8383	0.8480	0.8468 (0.0068)
Bilayer network (A)	0.9036	0.9029	0.9041	0.8890	0.8925	0.8984 (0.0071)

### 3.4 Experiments on two different datasets

Because the methods of NMF, CNMF, GNMFLMI, and SPCMLMI all belong to the matrix completion models, it is representative to put them together for comparison. In order to ensure that the prediction results are more convincing, we compared SPCMLMI with NMF, CNMF, and GNMFLMI under five-fold cross-validation on two different datasets (lncRNASNP dataset and lncRNASNP2 dataset), respectively. The lncRNASNP2 dataset was downloaded from http://bioinfo.life.hust.edu.cn/lncRNASNP (the January 2018 version) (Ya-Ru et al., 2018). After removing the duplicated entries, 8,634 experimentally confirmed lncRNA–miRNA interactions were obtained, including 262 miRNAs and 468 lncRNAs. As shown in Table 5, the AUC values of NMF, CNMF, GNMFLMI, and SPCMLMI on the lncRNASNP2 dataset were 0.9344, 0.9510, 0.9769, and 0.9891, respectively. We can see that the proposed method achieved the best performance. At the same time, the performance of our proposed method on the lncRNASNP dataset was also the best. We can see from Table 2 that the average AUC values of NMF, CNMF, GNMFLMI, and SPCMLMI on the lncRNASNP dataset were 0.8316, 0.8535, 8894, and 0.8984, respectively. The results further demonstrated that the method of SPCMLMI is effective and robust in predicting potential lncRNA–miRNA interactions.

**TABLE 5:** AUC values of SPCMLMI and other compared methods under five-fold cross-validation on the lncRNASNP dataset and lncRNASNP2 dataset.

Method	AUC values on lncRNASNP2	AUC values on lncRNASNP
NMF	0.9344 ± 0.0052	0.8316 ± 0.0080
CNMF	0.9510 ± 0.0054	0.8535 ± 0.0054
GNMFLMI	0.9769 ± 0.0022	0.8894 ± 0.0056
SPCMLMI	0.9891 ± 0.0020	0.8984 ± 0.0071

### 3.5 Case studies

In this section, case studies were performed on the lncRNASNP2 dataset to further validate the capability of SPCMLMI to infer novel lncRNA–miRNA interactions. In the experiment, we removed the interactions of a specific miRNA or the interactions of a specific lncRNA from the dataset and used the SPCMLMI method to predict lncRNAs interacting with “the specific miRNA” and miRNAs interacting with “the specific lncRNA.” We selected the lncRNA XIST (NONHSAT137542.2) and miRNA hsa-miR-195–5p as candidate prediction objects, respectively. The lncRNA XIST is closely related to non-small cell lung cancer and can promote cancer cell proliferation, invasion, and metastasis (Liu et al., 2019). The miRNA hsa-miR-195–5p has been proven to be a critical regulator in the progression of prostate cancer, which inhibits cell proliferation by downregulating proline-rich protein 11 expression (Cai et al., 2018). For the lncRNA XIST, all candidate miRNAs were sorted in descending order according to the predicted interaction scores after perturbing. The predicted top 10 candidate miRNAs interacting with the lncRNA XIST are shown in Table 6. We can see that seven out of them have been confirmed by biochemical experiments to be searched in starBase v2.0 and lncRNASNP2 databases. Similarly, for the miRNA hsa-miR-195–5p, we ranked all candidate lncRNAs according to their predicted scores in the perturbed matrix. As shown in Table 7, the top 10 candidate lncRNAs related to hsa-mir-195–5p were verified by biochemical experiments to be searched in starBase v2.0 and lncRNASNP2 databases. The aforementioned results further demonstrated the effectiveness of SPCMLMI in predicting novel interactions of miRNA with lncRNA.

**TABLE 6:** Top 10 candidate miRNAs for lncRNA XIST using SPCMLMI.

Rank	MiRNA	Confirmed?
1	hsa-mir-187–3p	NO
2	hsa-mir-411–5p	NO
3	hsa-mir-485–5p	YES
4	hsa-mir-653–5p	YES
5	hsa-mir-186–5p	YES
6	hsa-mir-544a	YES
7	hsa-mir-495–3p	YES
8	hsa-mir-137	YES
9	hsa-mir-128–3p	NO
10	hsa-mir-132–3p	YES

**TABLE 7:** Top 10 candidate lncRNAs for miRNA hsa-mir-195–5p using SPCMLMI.

Rank	LncRNA	Confirmed?
1	nonhsat055673.2	YES
2	nonhsat081836.2	YES
3	nonhsat039802.2	YES
4	nonhsat119666.2	NO
5	nonhsat117948.2	YES
6	nonhsat055703.2	YES
7	nonhsat081839.2	YES
8	nonhsat039834.2	NO
9	nonhsat117289.2	YES
10	nonhsat017523.2	NO

## 4 Discussion

As key molecules in the competing endogenous RNA (ceRNA) mechanism, lncRNAs and miRNAs play critical roles in gene regulation, and exploring their interactions shows a variety of biological functions. In this study, we developed a computational approach called SPCMLMI, which uses structural perturbation for matrix completion to infer lncRNA–miRNA interactions. We first make full use of the expression profiles and sequence information on lncRNAs and miRNAs to calculate their respective similarities. Then, according to the lncRNA similarity network, the miRNA similarity network, and the lncRNA–miRNA interaction network, we constructed the lncRNA–miRNA bilayer symmetrical network. Structural consistency was utilized to measure the link predictability of this network. The results suggested that the lncRNA–miRNA bilayer network achieved the best link predictability. Finally, we used the structural perturbation approach to perturb the bilayer network to recover the unknown links in the lncRNA–miRNA interaction network (i.e., to achieve the lncRNA–miRNA interaction adjacency matrix completion).

The performance of our method was compared with other competing methods on two different datasets. The experimental results demonstrated that SPCMLMI is powerful in predicting lncRNA–miRNA interactions. Although the results show that SPCMLMI is reliable and effective, there are some limitations. SPCMLMI only utilized two different miRNA/lncRNA-related pieces of information to construct the miRNA/lncRNA similarity network; we hope that more different miRNA/lncRNA-related information will be utilized to construct their similarity network in the future. Moreover, our method relies on the known lncRNA–miRNA interaction network. We look forward to building a more complete lncRNA–miRNA interaction network to improve the prediction performance by further studying lncRNAs and miRNAs.

## Data Availability

The original contributions presented in the study are included in the article/Supplementary Material; further inquiries can be directed to the corresponding authors.
